# Altered gut and adipose tissue hormones in overweight and obese individuals: cause or consequence?

**DOI:** 10.1038/ijo.2015.220

**Published:** 2015-12-01

**Authors:** M E J Lean, D Malkova

**Affiliations:** 1 Human Nutrition Section, School of Medicine, College of Medical, Veterinary and Life Sciences, University of Glasgow, Glasgow, Scotland

## Abstract

The aim of this article is to review the research into the main peripheral appetite signals altered in human obesity, together with their modifications after body weight loss with diet and exercise and after bariatric surgery, which may be relevant to strategies for obesity treatment. Body weight homeostasis involves the gut–brain axis, a complex and highly coordinated system of peripheral appetite hormones and centrally mediated neuronal regulation. The list of peripheral anorexigenic and orexigenic physiological factors in both animals and humans is intimidating and expanding, but anorexigenic glucagon-like peptide 1 (GLP-1), cholecystokinin (CCK), peptide YY (PYY) and orexigenic ghrelin from the gastrointestinal tract, pancreatic polypeptide (PP) from the pancreas and anorexigenic leptin from adiposites remain the most widely studied hormones. Homeostatic control of food intake occurs in humans, although its relative importance for eating behaviour is uncertain, compared with social and environmental influences. There are perturbations in the gut–brain axis in obese compared with lean individuals, as well as in weight-reduced obese individuals. Fasting and postprandial levels of gut hormones change when obese individuals lose weight, either with surgical or with dietary and/or exercise interventions. Diet-induced weight loss results in long-term changes in appetite gut hormones, postulated to favour increased appetite and weight regain while exercise programmes modify responses in a direction expected to enhance satiety and permit weight loss and/or maintenance. Sustained weight loss achieved by bariatric surgery may in part be mediated via favourable changes to gut hormones. Future work will be necessary to fully elucidate the role of each element of the axis, and whether modifying these signals can reduce the risk of obesity.

## Introduction

Projections suggest that, by 2030, obesity prevalence may reach over 45% of the entire US population, and 48% in the United Kingdom.^1^ Behind this lies a progressive rise in body fat of individuals with age, that is, the disease process of obesity, as recognized by the American Medical Association.^2^ By age 65, almost 40% of UK adults are now obese, and only 15% of men and 28% of women have a ‘normal' body mass index (BMI) of 18.5–25 kg m^−2^.^3^

Rapidly growing obesity research is now shedding light on the complex and interrelated biological and psychosocial underpinnings of appetite regulation and eating behaviour. The changing food environment and food culture have a major role in the recent rise in obesity: with ready availability of attractive high-calorie foods, often in excessive portion sizes, and sedentary lifestyles all now regarded as normal.^4^ However, a body of evidence supports a continuing role for the gut–brain axis in regulation of food intake and the maintenance of body weight.^5, 6, 7, 8^ Thus, a complex array of signals from peripheral and central nervous systems, possibly under epigenetic programming, interacts with psychological and social factors to determine energy balance and body weight homeostasis.^5^

A great deal of research has been applied to the search for genetic factors behind obesity. Although many single gene variants have been discovered, their individual and indeed cumulative effect sizes are rather small, and they do not appear to account for the dramatic epidemic rise of obesity internationally. Most appear to be associated with alterations in appetite/food intake, rather than metabolic effects on energy balance.^9^ It is possible that the rapidly growing study of epigenetics will reveal more mechanisms.

It is difficult to disentangle altered physiological factors that are possibly contributory, from the multiple biological consequences of weight gain and obesity. The underlying processes do not emerge only when people reach an arbitrary BMI or waist cutoff for ‘obesity', but exert causal influences from the earliest stages as body fat content begins to rise. The combined effects of physiological factors are usually only sufficient to generate weight gains of 0.5–1 kg year^−1^, which implies energy imbalances of around 10–20 kcal day^−1^, on average. This amount (<1% of metabolic rate and energy requirements)^10^ is too small to detect by calorimetric methods, but is enough to cause weight gains of up to 40–50 kg over an adult life.^11^ These considerations indicate the significant difficulties that face any attempt to provide evidence for a quantitative mechanistic physiological explanation underlying obesity.

Over the past 20 years, there has been a glut of information on the physiological control of appetite in animal models; more than 30 gut hormones, neuropeptides and neurotransmitters are now known to affect appetite (Table 1). Many have been documented in both animals and humans, so might have roles in developing or treating obesity. Pharmacological agents directed at the compounds involved in physiological control of appetite have increasingly been investigated.^12^ However, even a drug that could completely reverse one major mechanism affecting appetite may not be expected to have an effect on body weight greater than perhaps 5–10%^13^ when used as monotherapy, as other mechanisms remain active, and because beyond young childhood humans eat in response to a wide variety of physical or sensory cues and social triggers.^14^ The experience of eating for pleasure, akin to addictive behaviours (rather than appetite), is commonplace for most people even without obesity, and eating as a result of boredom is also very common. Social marketing by the food industry has exploited this to great advantage.

The gut–brain axis is the physiological driver of satiation in humans. Meal ingestion results in gastric distension and production of peptide hormones by enteroendocrine cells, both of which can promote a feeling of fullness/satiety, and a desire to stop eating; this topic has been extensively reviewed elsewhere.^15, 16, 17^ Peptide hormones generated in the gastrointestinal (GI) tract and adipose tissue modulate appetite in both animal and human studies (Table 1). Peptides released, in response to ingested food, from multiple sites in the gut (including the stomach, proximal/distal small intestine, pancreas and colon), activate vagal afferent nerves that innervate brain regions involved in the immediate need for food intake.^18, 19^ Peripheral signals from adipose tissue and the gut are integrated in the hypothalamus to influence short-term food intake and long-term energy balance; centrally released hormones and neurotransmitters also participate in appetite regulation.^20, 21, 22^ Cross-talk between peripheral satiety signals and hypothalamic and brainstem centres represents an integrative regulatory system for feeding, energy balance and body composition.

Homeostatic and hedonic systems in the brain also influence appetite and satiety. The physiologic *need* to eat during negative energy balance is a primitive survival function, mediated through the hindbrain and hypothalamus.^5, 23^ In contrast, a *desire* to consume attractive foods arises from mesolimbic reward circuits and regions in the orbitofrontal cortex that regulates hedonic food intake based on the sensations of taste, smell, texture and sight.^23, 24, 25^ The hedonic systems are in constant interaction with homeostatic mechanisms and sensory attractiveness of foods can become dominant to contribute to weight gain and obesity. These mechanisms cannot be considered totally separate, indeed gut hormones such as ghrelin can alter the hedonic perceptions of food on presentation and consumption in response to negative energy balance, perhaps via the FTO gene expression or through dopaminergic mechanisms.^26^ A possible partial explanation for the recent obesity epidemic is that sensory stimulation related to food (that is, hedonic inputs) and overconsumption of palatable, energy-dense meals has increased markedly in society, whereas basic homeostatic satiety controls have remained stable.^27, 28^

The concept of a physiological ‘set point' whereby energy intake is automatically adjusted to maintain weight, or a specific component of body composition, is now relatively old, but begs questions about mechanisms. New evidence may suggest that there are structural changes in the hypothalamus, which could be responsible for resetting the putative set point in such a way that appetite will defend an increased body weight.^29^ The extent to which this might be relevant outside cases of hypothalamic obesity is uncertain, and there remains evidence that some people can adapt to live with a lower body weight after intentional weight loss.

This review aimed to provide a listing and concise summary of the hormones currently recognized to affect appetite, as a reference point for clinicians and researchers. It followed a structured approach to reach the totality of the published literature, to examine the currently available evidence on the biological regulation of appetite and the dysregulated gut- and adipose tissue-derived hormone signalling in overweight and obese individuals. It evaluates evidence on peripheral hormone responses to weight loss, achieved by either dietary and exercise interventions or surgical procedures, and aims to identify potential links between changes in hormone levels and obesity with a view to determine which changes are secondary and which may be primary and potentially causal.

## Materials and methods

Studies were identified by searching PubMed and Embase electronic databases for peer-reviewed, English-language publications between June 2008 and July 2013. Original studies were identified from the two databases by searching for the following terms in the title or abstract: ‘appetite', ‘satiety', ‘satiation', ‘satiety response' or ‘post-meal satiety' in conjunction with: ‘obesity', ‘body fat', ‘weight gain', ‘weight reduction', ‘weight loss', ‘waist circumference' or ‘body mass index' and together with: ‘hormone(s)', ‘peptide(s)', ‘glucagon-like peptide 1', ‘peptide YY', ‘leptin', ‘ghrelin', ‘pancreatic polypeptide', ‘obestatin' or ‘cholecystokinin'. Approximately 600 papers were identified and reviewed for relevance by two reviewers. Original studies were chosen for inclusion if they contained direct comparisons of hormonal satiety responses to a test meal in overweight or obese subjects versus lean controls. Studies of hormonal satiety responses before and post gastric bypass surgery or other weight loss interventions were included, as were neuroimaging studies that examined the effects of obesity on brain activation. The examination of bibliographies from published papers identified important sources published earlier than 2008.

There is a large body of literature on obesity and satiation in animals; articles were selected from the search results to illustrate the hormones, peptides and neurotransmitters listed in Table 1, with additional focused searches conducted to confirm the level of evidence (where 0=animal studies only, 1=observational clinical studies, 2=randomized controlled trials, 3=systematic reviews of observational studies and 4=systematic reviews of randomized controlled trials (with or without additional observational studies)). A number of peptides have not been included in the present review. For example, tumour necrosis factor was excluded because the subject population in most studies comprised patients with cancer or undergoing haemodialysis. Pro-opiomelanocortin was also excluded, as it is a precursor for adrenocorticotropic hormone and α-melanocyte-stimulating hormone, both of which are included in this review. Some additional peptides originally thought to influence eating habits no longer fit into either anorexigenic or orexigenic categories (for example, adiponectin has evidence for both; obestatin was originally thought to be anorexigenic, but more recent studies have not confirmed this and even suggested that the activity of obestatin may be dependent on ghrelin^30^). When reviewing Table 1, note that preclinical models are not entirely analogous to obesity in humans, as stress-induced changes in feeding behaviour may result from different animal handling methods.^16, 31^ Food intake by humans is, in large part, influenced by external factors and cognitive processes that are not operative in animals.^5, 27^ Thus, although there is an ongoing contribution of animal studies towards better understanding of the mechanisms underlying obesity, this review focuses primarily on findings from studies in humans, with a view to establishing which alterations in mechanisms are secondary consequences of excess adiposity, which may be causal, and which may retain some potential for exploitation in obesity treatment.

### Obesity and dysregulated appetite signalling

Findings from studies comparing the effects of obesity in humans on concentrations of peripheral appetite hormones in the fasting state or following a test meal and evidence on peripheral hormone responses to weight loss induced by dietary and/or exercise interventions or surgical interventions are reviewed. Emphasis has been placed on the hormones that have been the most widely studied (Table 1). In research of this kind, it is important to distinguish between dynamic effects arising from acute negative energy balance, and static effects that reflect a change in body composition. It is therefore important to include a period of weight stability after weight loss to remove the effects of acute energy imbalance, while recognizing that the components of energy balance may be different during weight stability (energy balance) after weight loss, for example, there may be altered macronutrient composition of the diet, or greater physical activity.

It is implicit in any analysis of endocrine functions that hormone actions must always reflect both circulating concentrations and also receptor number and sensitivity. In many cases, while it is relatively straightforward to measure plasma concentration, and sometimes circulating receptors, it is usually impossible to assess receptor or post-receptor functions *in vivo*. Sensitivity or resistance to a hormone action is invoked empirically when there are different responses to the same concentration.

#### Glucagon-like peptide 1

Glucagon-like peptide (GLP-1) (Table 1) is the product of post-translational processing of pro-glucagon in the gut and the brain.^32^ In the gut, GLP-1 is secreted primarily from enteroendocrine L cells located in the distal jejunum and ileum, and its release is stimulated by carbohydrate and fat intake.^33, 34^ GLP-1 receptors are widely distributed in the brain, in peripheral organs such as the pancreatic islets, and in the whole GI tract. In the brain, GLP-1 receptors are found in areas that are implicated in the control of food intake and energy balance. GLP-1 has several other physiological functions: it acts as a strong incretin, that is, stimulates glucose-induced insulin release, and it inhibits glucagon release.^35, 36^ In addition, GLP-1 activates the ‘ileal brake' by slowing the rate of gastric emptying, which slows nutrient absorption and contributes to the reduction of postprandial glycaemia and enhanced satiety,^34, 35^ and may explain nausea as a transient effect of GLP-1 receptor agonists.^37^ Both central and peripheral administration of GLP-1 have been shown to reduce food intake. Secretion of GLP-1 in response to food intake is biphasic: the early phase occurs within 10–15 min after food ingestion with a second peak at 30–60 min.^34^ After release, GLP-1 is rapidly inactivated and has a plasma half-life of less than 2 min.

**Figure 1:**
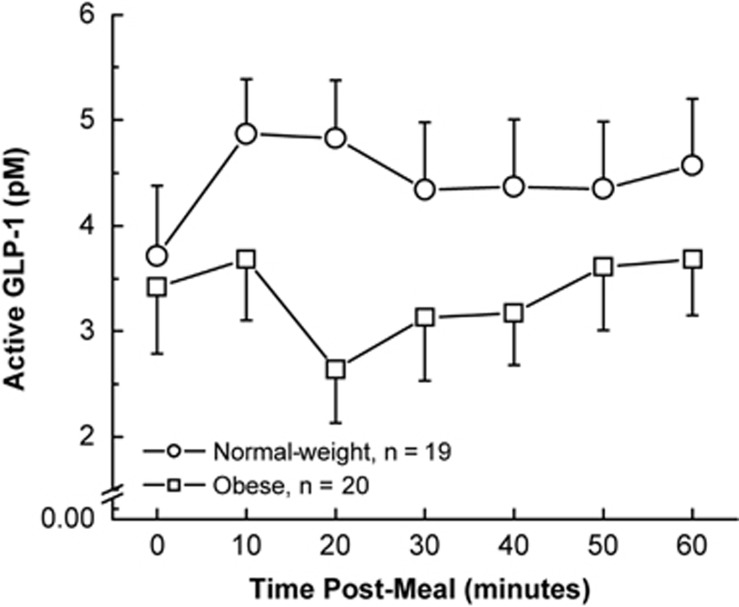
GLP-1 responses to a liquid test meal in 19 normal-weight and 20 obese subjects.^39^ Reproduced with permission.

Obesity has been associated with an attenuated postprandial GLP-1 response to test meals in a number of cross-sectional and longitudinal studies.^38, 39, 40^ Preprandial GLP-1 levels were similar for overweight/obese adults compared with normal-weight controls in a study conducted by Adam and Westerterp-Plantenga,^38^ but the postprandial GLP-1 response was significantly blunted in overweight/obese subjects 30 min after a test meal compared with control subjects. Another study revealed that while in normal-weight subjects GLP-1 levels increased 10 min after a standard liquid meal, in obese subjects GLP-1 levels declined markedly in the first 20 min (Figure 1).^39^ These data, however, contrast with a similar study by Bowen *et al.*^41^ in which postprandial GLP-1 levels were significantly greater in obese versus lean subjects after consumption of a buffet lunch. It should be noted that in this study the fasting concentrations of GLP-1 were also significantly higher in the obese individuals. Thus, the postprandial response may have been influenced by the fasting GLP-1 concentration rather than by obesity status.

Studies have examined the effects of dietary interventions for weight loss on GLP-1 levels. After a reduction of ⩾8% of an initially obese body weight, with an 8-week low-energy diet (1000 kcal day^−1^) and a 2- to 3-week control diet standardization period, lower levels of fasting GLP-1 and greater appetite were observed compared with assessments in the same patients after a 6-month high monounsaturated fat dietary intervention which was accompanied by a modest (3.6 kg, 4.2%) weight regain.^42^ Another study monitored patients after an 8-week very-low-calorie diet (500–550 kcal day^−1^), followed by a 2-week weight stabilization period and a 52-week weight maintenance phase.^43^ After weight stabilization at week 10, patients had a mean weight loss of 14% of initial body weight. Patients regained some weight during the maintenance phase, remaining a mean of 8% below their initial body weight at week 62. Postprandial GLP-1 levels after 62 weeks were significantly lower than at baseline. The above studies support the view that modest diet-induced weight loss can result in long-term reductions in GLP-1, postulated to favour increased appetite and weight regain. This may at least in part explain why weight loss through caloric restriction is often so difficult to achieve and/or maintain for obese individuals.

Although caloric restriction-induced weight loss reduces GLP-1 levels, weight loss with exercise was reported to induce a response in the opposite direction. Martins *et al.*^44^ were the first to examine the effect of aerobic exercise training on fasting and postprandial levels of GLP-1 in obese individuals; a 12-week supervised exercise training programme (500 kcal of treadmill walking or running 5 days per week) had no impact of fasting GLP-1 concentration, but tended to increase its postprandial release.^44^ The tendency for a beneficial change in GLP-1 levels may explain why long-term exercise training typically permits body weight reduction and helps maintenance. The recent meta-analysis revealed a small mean effect for acute exercise to increase GLP-1 levels in normal-weight individuals.^45^ It remains unclear whether these findings apply to obese and overweight individuals.

#### Peptide YY

Peptide YY (PYY) is a satiety hormone whose anorexigenic effects are attributed to delayed gastric emptying (that is, the ileal brake) that is dose related and dependent on the amount of fat in the meal.^46^ PYY is co-secreted with GLP-1 by L cells in the lower intestine.^47, 48^ Postprandial levels of PYY peak within 2 h of eating and are proportional to the size and type (fat>protein>carbohydrate) of the ingested meal.^49, 50^ Fat intake is the strongest stimulant of PYY secretion, whereas carbohydrate intake has a limited effect in obese or non-obese individuals.^51, 52^

**Figure 2:**
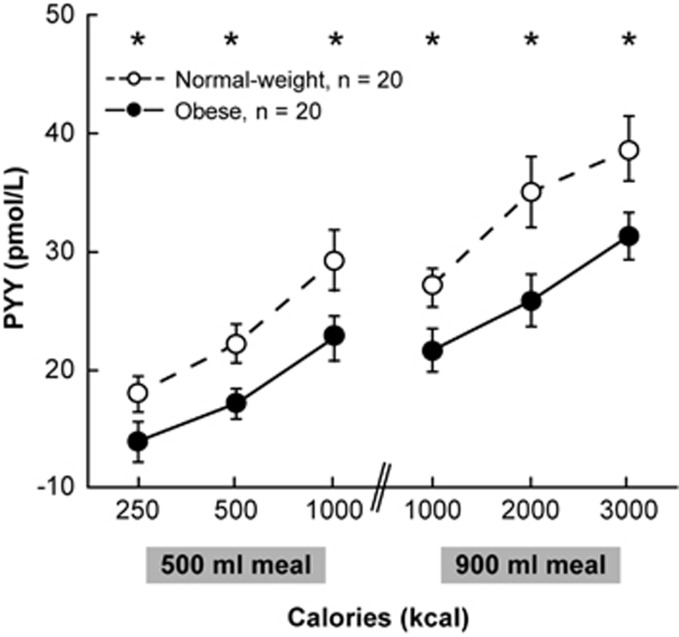
PYY responses 90 min after each of six test meals of increasing caloric content in 20 obese and 20 normal-weight subjects. *P<0.05 (unpaired *t*-test).^49^ Reproduced with permission.

Several studies have documented the obesity-related attenuation of postprandial responses in PYY levels.^53, 54, 55^ For example, in one study, the PYY response in obese subjects was significantly lower than in normal-weight controls after each of six test meals of increasing caloric content (Figure 2). This attenuated response corresponded with significantly lower subjective ratings of fullness in obese subjects, beginning 30 min after the meal and persisting for 3 h.^49^ Zwirska-Korczala *et al.*^55^ also observed blunted postprandial PYY responses in obese and morbidly obese women compared with lean control subjects. Racial differences in PYY responses were examined in another study of obese and lean women.^53^ Significantly lower postprandial PYY levels were noted in obese black women compared with lean black women, lean white women or obese white women; unfortunately, given the known racial differences in body compositions, these authors did not report body fat content or any relationship between PYY levels and body composition.

Similar to the findings with GLP-1 levels, in obese subjects weight loss of ⩾8% from an 8-week low-energy diet followed by a 2- to 3-week weight stabilization period resulted in decreased levels of fasting PYY and increased hunger.^42^ Short-term diet-induced weight loss in another study also resulted in significant reductions in postprandial PYY levels and a significant increase in subjective appetite that persisted over a 12-month weight maintenance phase.^43^

In contrast to dietary intervention studies, exercise studies conducted on overweight and obese individuals have reported increases or no change in PYY levels. An increase in fasting plasma PYY concentrations after 32 weeks of exercise training occurred in overweight male and female adolescents who experienced a significant decrease in body fat.^56^ Similarly, a significant increase in fasting PYY concentrations was reported in obese children who lost body weight following a 1-year diet and exercise intervention.^57^ One report noted a tendency for increased postprandial PYY concentrations in overweight and obese men and women after a 12-week exercise intervention.^44^ More recently, a study by Guelfi *et al.*^58^ including overweight and obese men found that neither endurance nor resistance training significantly altered fasting and postprandial levels of PYY despite aerobic training being associated with increased satiety.^58^ Thus, evidence on the contribution of PYY to improved satiety during weight loss with exercise training remains controversial. The impact of a single exercise session on PYY response is more obvious, with most acute studies reporting a small increase in PYY levels immediately after exercise.^45^ This increase is consistent with the notion that acute increases in PYY can be expected to induce suppression of hunger and potentially diminish compensation for the expended energy. To date, no studies have been published investigating the impact of acute exercise on peptide YY responses in overweight and obese individuals.

#### Pancreatic polypeptide

Pancreatic polypeptide (PP) is produced under vagal control by the peripheral cells of the endocrine pancreatic islets, and to a lesser extent in the exocrine pancreas, colon, and rectum in response to a meal and to insulin-induced hypoglycemia (Table 1).^16, 59, 60^ In humans, administration of pharmacological doses of PP has been shown to decrease food intake.^22^ Furthermore, PP inhibits the gastric emptying rate, exocrine pancreatic secretion and gallbladder motility.^60^ Studies of PP in obese humans have demonstrated conflicting results. Some studies found no difference between lean and obese subjects,^61^ whereas other studies demonstrated lower fasting PP levels in obese subjects.^62^ If PP levels are decreased in obese subjects, then this could reflect a consequence of being overweight or, on the other hand, be a cause of being overweight as PP affects food intake.

Evidence of the effect of weight loss on PP concentrations in obese individuals is inconsistent.^42, 43, 62, 63^ In one study, both postprandial PP concentrations and subjective ratings of hunger were increased after diet-induced weight loss and stabilization; the alterations persisted for at least 1 year after the initial weight loss.^43^ An increase in PP concentration following weight loss was also found in obese children.^62^ Thus, it could be assumed that the direction of change in PP following diet-induced weight loss is different from that reported in other gut hormones. However, fasting levels of PP, as with other anorexigenic gut hormones measured, were found to be reduced and appetite scores were increased in obese patients who experienced weight loss (in response to a short-term, low-energy diet) and weight stabilization compared with the same patients after they experienced some weight regain. In addition, postprandial levels of the gut hormone increased compared with fasting, but significant differences between before and after weight regain were not apparent.^42, 43^

Literature regarding the impact of exercise on PP is scarce and limited to acute exercise studies. As with GLP-1 and PYY, the recent meta-analysis found that a single exercise session increases the area under the PP concentration versus time curve.^45^ This exercise session-induced increase in PP can potentially contribute to appetite reduction reported during the hours immediately after exercise.

#### Cholecystokinin

Cholecystokinin (CCK) is produced in duodenal and jejunal ‘I' cells and secreted in response to luminal nutrient (particularly fat) intake (Table 1).^22, 64, 65^ Structurally, CKK is related to gastrin and it exists in numerous molecular forms with different numbers of amino acids, for example, CKK-8, CKK-33 and CKK-39.^66, 67^ The main effect of CCK on the GI system is to facilitate nutrient absorption, stimulate gall bladder contraction, enhance pancreatic enzyme secretion and slow gastric emptying.^68, 69, 70^ Delayed gastric emptying has been considered as a central mechanism by which CCK suppresses appetite.^71^ CCK is also present in different parts of the brain such as the amygdala, cortex, hippocampus, thalamus, hypothalamus, septum, dorsal hindbrain and basal ganglia, acting as a neurotransmitter.^67, 69, 72^

Compared with baseline measures, mean postprandial plasma concentrations of CCK were reduced in overweight and obese subjects who had lost a mean of 14% of their of initial weight after an 8-week very low-energy diet and a 2-week weight stabilization phase.^43^ Reductions in CCK levels persisted during the 1-year weight maintenance period. Similarly, rapid weight loss of approximately 15% of body weight in obese men who participated in a very-low-calorie diet intervention for 8 weeks and 1 week of weight stabilization resulted in significantly decreased postprandial CCK concentrations compared with baseline.^73^ These two studies suggest that CCK is reduced following rapid weight loss, a mechanism that might favour increased appetite and weight regain, and thus have a survival value. Both studies included a weight stabilization phase of 1–2 weeks; however, it is not certain whether this is sufficient for full metabolic adaptation to a neutral energy balance in overweight and obese subjects.

Evidence regarding the effect of exercise on CCK concentration, as with PP, is very limited. The recent study by Martins *et al.*^74^ reported that a 12-week exercise programme undertaken by overweight and obese sedentary individuals induced an average 3.5 kg weight loss but had no significant effect on either fasting or postprandial concentrations of CCK measured at least 48 h after the last exercise session to exclude the acute effects of exercise. The same study also found that exercise improved the ability to differentiate between preloads with different energy content. This implies that modifications in eating behaviour commonly seen with exercise are probably related to changes in levels and/or sensitivity of other appetite-regulating hormones and, as discussed in Supplementary information, alterations in neuronal responses to food. So far, data on the impact of acute exercise on CCK levels are available only from studies conducted on normal-weight individuals. These studies have reported increased CCK responses immediately after exercise and for up to 2 h.^75, 76^ This significant increase in CCK concentrations observed in acute exercise studies is in line with suppressed hunger feelings reported during the immediate hours after exercise.^77^

#### Leptin

Leptin is a key adiposity hormone produced tonically by mainly white adipose tissue. Secretion increases as lipid content rises and it signals to receptors in the hypothalamus to reduce appetite and increase energy expenditure (Table 1).^78^ The main physiological role of leptin is probably through its suppression, during starvation and thinness, to increase appetite; when normal body fat content is restored, leptin is again secreted. Leptin also has a role in promoting fertility.^79^ Recent evidence suggests that leptin potentiates the effects of CCK on inhibition of food intake and that this interaction is disrupted in obesity.^80^ Humans with rare mutations in the leptin or leptin receptor gene suffer extreme early-onset obesity and have endocrine defects.^81, 82^ Obesity and other consequences of leptin deficiency are corrected by long-term replacement therapy, so it appears certain that leptin has a key function in the regulation of energy balance in humans. However, in studies of adult humans, leptin concentrations correlated with total fat mass and appear to have little influence on energy intake. Leptin levels are decreased in patients with anorexia nervosa in a state of semi-starvation; during refeeding, levels increase in a BMI-dependent manner.^83^

In one study of lean and severely obese women, leptin levels were measured after a 12-h fast (baseline), and then over a 2-h period (measured at 0, 15, 30, 60, 90 and 120 min) following ingestion of a body weight-adjusted test meal (approximately one-quarter of the woman's kcal needs; range, 576.1–803.8 kcal; 60% carbohydrate, 20% protein and 20% fat).^84^ Baseline leptin levels were significantly higher in the obese subjects at baseline. These levels decreased gradually over the first 90 min after the test meal and precipitously thereafter (at 120 min). This is in contrast to lean subjects in whom leptin levels remained relatively constant over the 2-h postprandial period. In another study, baseline leptin levels were also higher in obese subjects compared with lean controls; levels did not change in either group during the first 60 min following a test meal of up to 510 kcal.^39^ Higher circulating concentrations of baseline leptin in obese individuals, with greater fat masses, are not surprising. However, because leptin promotes satiety, these studies support the notion that obese individuals exhibit some type of central leptin resistance that disrupts the satiety response. In two studies, diet-induced weight loss of approximately 15% of initial body weight in obese subjects resulted in fasting leptin levels that were 35% lower than baseline and remained reduced compared with baseline for up to 1 year after weight loss and stabilization.^43, 85^ The role of leptin in satiety was further examined in obese subjects who underwent a weight reduction programme and stabilization at 10% below their initial weight.^86^ Subjects received subcutaneous ‘replacement doses' of recombinant human leptin or placebo twice daily for 5 weeks each. After ingesting a liquid-formula diet, subjects reported significantly higher levels of satiation on a visual analogue scale following leptin than following placebo injections. These studies further support the role of leptin in the regulation of satiety and suggest that leptin resistance might to some extent be overcome by massive weight loss. A role for physical activity, permitting transport of leptin into the brain, has also been proposed as a way to overcome leptin resistance.^87^

The notion that participation in exercise improves the activity of leptin is supported by evidence that exercise, when associated with mass loss, is followed by a significant reduction in fasting leptin concentrations and that this change increases rather than reduces subjective hunger measured in the fasted state.^88^ A recent study reported that exercise-induced weight loss leads not only to a reduction in fasting, but also in postprandial, concentrations of leptin.^74^ Another study involving a 12-week supervised exercise programme in sedentary overweight and obese individuals suggested that leptin concentrations measured both in the fasted state and after high- and low-energy test meals were significantly lower following exercise intervention, resulting in up to approximately 3.5 kg of weight loss.^74^ Despite this, the accuracy of compensation for previous energy intake was improved. Thus, reduced leptin concentrations after chronic exercise might indeed reflect improved leptin action.

#### Ghrelin

Ghrelin is a 28-amino acid peptide originally identified as the endogenous ligand of the growth hormone secretagogue receptor 1a (GHS-R1a);^89^ besides having growth hormone-releasing activity, it is a potent orexigen with a fundamental influence on appetite and energy homeostasis regulation.^7, 18^ Ghrelin is primarily expressed in the stomach as well as in the hypothalamus. The orexigenic functions of ghrelin have been attributed to total and acylated ghrelin, while unacylated form (>90% of total ghrelin) has been considered as a non-functional peptide.^90^ Ghrelin concentration peaks in response to fasting and anticipation of the coming meal,^91^ and thus initiates and promotes eating (Table 1). Concentrations in normal-weight individuals exhibit a preprandial peak with a decline after eating.^92^ Working in opposition to satiety hormones, ghrelin increases the rate of gastric emptying^93^ and increases hunger.^94^ However, some recent data do not favour a role of peripheral ghrelin in the regulation of food intake.^95, 96^ First, ghrelin infusion at both physiological and supraphysiological doses was found to have no effect on appetite or spontaneous meal request.^95^ In addition, it has been reported that food restriction-induced increases in appetite were not influenced by changes in plasma acylated ghrelin concentrations. At the same time over the past few years, effects of unacylated ghrelin on insulin sensitivity, metabolism, muscle regeneration and β-cell protection have been reported.^97, 98^ Regardless of the current debate about the primary function of ghrelin, research investigating the role of this hormone in appetite regulation deserves discussion.

**Figure 3:**
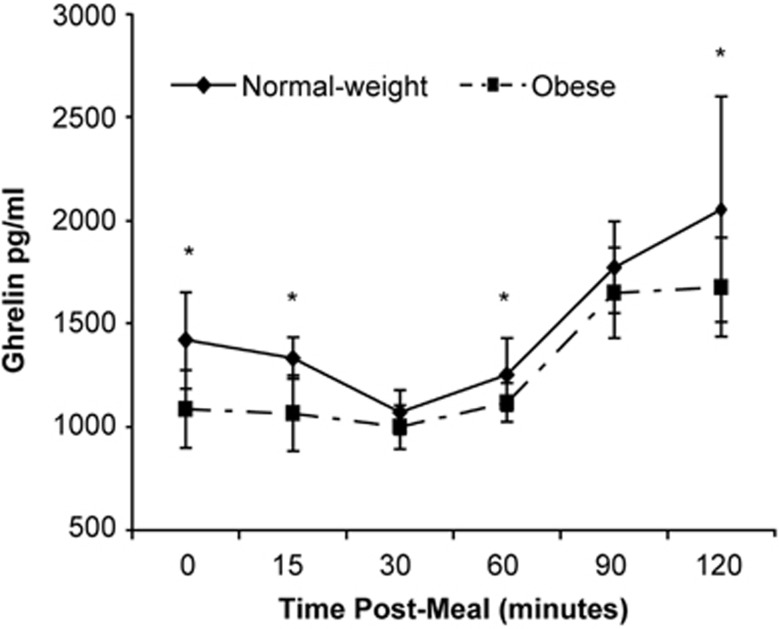
Ghrelin responses to a test meal in 10 normal-weight and 13 severely obese women. Ghrelin levels were significantly higher. *(*P*⩽0.05) in normal-weight women at baseline (*P*=0.001) and at 15 (*P*=0.001), 60 (*P*=0.032) and 120 (*P*=0.044) min postprandially.^84^ Reproduced with permission.

Fasting and postprandial plasma ghrelin levels were reported to be lower in obese than in normal-weight individuals (Figure 3).^84^ Little change from fasting levels was noted in obese subjects in other studies.^39, 50, 55, 84, 85, 99^ One of these studies demonstrated a 30% reduction from preprandial ghrelin levels in lean women, beginning 30 min after a test meal, but no significant changes were noted in either obese or severely obese women.^55^ There may be nutrient-specific effects on ghrelin levels. Yang *et al.*^50^ found greater postprandial suppression of ghrelin in obese and lean subjects following a high-carbohydrate meal (88% carbohydrate, 8% protein and 4% fat) than after a high-fat meal (25% carbohydrate, 4% protein and 71% fat). However, compared with lean subjects, obese subjects demonstrated less suppression of ghrelin following either meal.

Thus, observations of the relationship between obesity and failure to suppress ghrelin levels after a meal are consistent across studies. The flattened hormone profiles around traditional mealtimes may be a factor in the continuous ‘grazing' pattern reported in obese subjects.^100, 101^

Diet-induced weight loss in obese subjects was accompanied by alterations in ghrelin levels in several studies. Compared with baseline measures, plasma ghrelin was increased by 17% in overweight and obese women who reduced their body weight by 4.5% at the completion of a 10-week weight loss intervention programme that included an energy-restricted diet of 40% carbohydrates, 30% fat and 30% protein.^102^ It should be noted that these results were obtained at the end of the acute intervention, and thus participants were presumably still in a state of negative energy balance, which may have confounded the results. In two other studies, short-term diet-induced weight loss in obese subjects resulted in an exaggerated pattern of postprandial changes in ghrelin levels compared with the pre-weight loss setting, with significantly greater levels persisting over weight maintenance/stabilization periods of 6 and 12 months.^43, 85^ Repeated weight loss and regain may result in long-term alterations in ghrelin signalling. Among 159 weight-stable obese or overweight women, a history of intentional weight loss over the preceding 20 years, defined as episodes of intentionally losing at least 10 pounds, and frequency of intentional weight cycling were associated with higher fasting levels of ghrelin, in analyses adjusted for age, BMI and physical activity.^103^ This evidence raises the intriguing possibility that, as well as weight loss, weight cycling may promote an appetite-stimulating hormonal profile, further hindering weight maintenance. However, reaching this conclusion hinges on effective adjustment of the data for confounders, of which the most important is body composition; relatively small differences in body composition may introduce errors, and BMI is not sufficiently specific to avoid these errors.

The role of ghrelin in the control of appetite has been examined extensively in exercise studies. A recent meta-analysis reported that in normal-weight individuals, acute exercise suppresses acylated ghrelin levels,^45^ in line with a transient suppression of hunger in the hours immediately after exercise.^104^ However, chronic exercise resulting in significant weight loss was found to enhance fasting concentrations of total^105^ and acylated^44^ ghrelin in obese and overweight individuals. At the same time, increased suppression of postprandial acylated ghrelin was observed in one of the studies.^44^ This is in line with the observation that although medium-term exercise-induced weight loss increases both fasting hunger and hunger across the day, it also improves meal-induced satiety.^106^

In contrast to the above results, a study conducted in a group of morbidly obese men and women showed that fasting and meal related circulating ghrelin levels remained unchanged despite 5% weight loss induced by a 3-week integrated body weight reduction programme with exercise training.^107^ No impact of aerobic training on circulating acylated ghrelin levels was found in a study conducted on overweight and obese men^58^ and overweight children.^108^ Differences in fat mass loss, volume and duration of exercise interventions, and inclusion of different genders are likely to be factors contributing to the discrepancy. In addition, changes in ghrelin concentration with exercise may be also dependent on extent and direction of change in levels of other potential appetite regulators, such as a leptin, insulin and probably PYY.^109, 110^

### Effect of corticosteroids on gut peptides

Elevated corticosteroid status provides a useful model for some types of obesity. Weight gain varies widely in human clinical studies, depending on dose/circulating concentration and on individual factors. Alterations in the hypothalamic–pituitary–adrenal axis have been demonstrated in obesity.^111^ Weight gain is a feature of Cushings syndrome and a common side effect of glucocorticoid therapy. A systematic review has suggested an average weight gain of 2 kg, but the stringent inclusion criteria of that review resulted in only one RCT paper being included.^112^ It is surprising that weight change with corticosteroids, which can often be large, has not been better documented. Therapeutic doses of glucocorticoids were shown to increase food intake in healthy male volunteers (*N*=10).^113^ Vicennati *et al.*^114^ reported weight gain and the development of obesity in women (*N*=14) after a significant stressful event; these changes were manifested via a hyperactive adrenal cortex.^114^ In another study, stress-related cortical secretion was positively correlated with BMI, waist-to-hip ratio and sagittal recumbent trunk diameter in a population of 51-year-old men (*N*=284).^115^ In a study examining the effects of methodology on gut hormone levels in 10 normal-weight men, the temporal plasma PYY concentration profile was altered by study-induced stress, and the PYY area under the curve was positively correlated with the cortisol area under the curve.^116^ In 33 young adults, serum ghrelin exhibited a strong inverse association with serum cortisol during 12–84 h of fasting (*r*=−0.79; *P*<0.0001).^117^ However, in a separate study, prednisone treatment (30 mg day^−1^ for 5 days) significantly suppressed plasma ghrelin levels by a median of 18.3% in eight healthy volunteers; thus, the authors concluded that weight gain observed with glucocorticoid treatment may not be mediated by ghrelin.^118^

## Conclusions

Obesity in humans arises from excess energy consumption relative to expenditure over long periods, with secondary hyperphagia that opposes weight loss. Although adult eating patterns are influenced substantially by social, cultural and commercial factors, food intake is also regulated by a complex and inter-connected array of peripheral hormonal signals, originating in the gut and adipose tissue in humans.

The list of peripheral hormones that regulate appetite and food intake is long and growing. Studies related to gut and adipose tissue hormones and obesity suggest that obesity is associated with a blunted postprandial response of satiety factors such as GLP-1 and PYY, CCK and reduced postprandial suppression of orexigenic ghrelin, and some type of central leptin resistance. Caloric restriction-induced weight loss causes persistent changes in peripheral hormones that tend to increase appetite and promote weight regain, while bariatric surgery and exercise programmes modify responses in a direction expected to enhance satiety and permit weight loss and/or maintenance. This suggestive causal relationship between weight loss following caloric restriction, exercise and gastric bypass surgery (see Supplementary information) and peripheral hormone alterations was not found in all studies.

Research into the regulation of appetite and body weight has seen a dramatic increase in the last 20–30 years, as the obesity epidemic has emerged, and the recognition of adipose tissue as an active endocrine organ is one of the most remarkable paradigm shifts of the last 100 years. Future research must focus clearly between causal factors in the *disease process* of obesity and physiological changes and adaptations to the state of obesity. Simply measuring differences between obese and non-obese individuals can be misleading. To unravel and begin to correct the causes of the obesity epidemic there is a need for more studies on longitudinal changes, and on the interactions between potential factors in this complex, multifactorial disease. The principle drivers of the epidemic seem clearly to be social, with both reduced physical activity and greater provision and promotion of energy-dense foods contributing. It is now incontrovertible that endocrine factors are playing a part, but the extent to which their roles are causal needs definition. The extent to which interventions via endocrine systems can ameliorate the situation for obese people will be defined by ongoing and planned clinical research, but modern analogue therapies appear to offer remarkably low-risk and effective treatments when used in conjunction with nutritionally-sound evidence-based diet and exercise regimens.

Taken together, the research findings presented in this review are building a foundation for the identification of targets for life style and therapeutic interventions in obesity. Ongoing research will further our understanding of the effects of obesity on body weight homeostasis and potentially enable us to exploit, at least for some obese individuals, the effects of gut and adipose tissue hormones involved in the regulation of appetite.

## Figures and Tables

**Table 1:** Hormones, neuropeptides and neurotransmitters involved in appetite regulation based on animal and/or clinical studies

*Hormone, neuropeptide, neurotransmitter*	*Primary source for appetite regulation*	*Proposed mechanism of action on appetite*	*Level of evidence*
*Anorexigenic factors*			
α-Melanocyte-stimulating hormone (α-MSH)	Hypothalamus	Stimulates hypothalamic MC3/4 receptors	2^(ref. 119)^ (1 RCT)
Acetylcholine	CNS; PNS	Nicotinic acetylcholine receptor-mediated modulation of hypothalamic circuits regulating appetite and cortico-limbic-striatal reward circuits	2^(ref. 120)^ (3 RCTs)
Adrenocorticotropic hormone (ACTH)	Hypothalamus	Stimulates hypothalamic MC3/4 receptors	2^(ref. 119)^ (4 RCTs)
Amylin	Pancreatic β cells (co-secreted with insulin)	Delays gastric emptying; targets the hindbrain and hypothalamus	4^(ref. 121)^ (5 RCTs)
Apolipoprotein A-IV	Intestinal epithelial cells	Inhibits gastric motility, targets the hypothalamus	1^(ref. 122)^
Catecholamines (dopamine, epinephrine, norepinephrine)	Brain stem, adrenal gland	Acute decrease in food intake	2 ^(ref. 123)^ (14 RCTs)
Cocaine- and amphetamine-regulated transcript (CART) peptide	Hypothalamus	Modulates hypothalamic and hindbrain circuits	1^(ref. 124)^
Cholecystokinin (CCK)	Duodenal/jejunal I cells	Delays gastric emptying; targets the vagus nerve, hindbrain, and hypothalamus	2^(ref. 41)^ (37 RCTs)
Enterostatin	Exocrine pancreas	Inhibits fat intake, targets the hypothalamus	2^(ref. 125)^ (1 RCT)
Gastrin-releasing peptide (GRP)	Gastric myenteric neurons	Contributes to meal termination	2^(ref. 126)^ (1 RCT)
Glucagon	Pancreatic α cells	Modulates gastric emptying and vagal tone	4^(ref. 127)^ (large number of RCTs, although these may include studies of GLP-1)
Glucagon-like peptide 1 (GLP-1)	Intestinal L cells (co-secreted with PYY, OXM)	Decreases gastric emptying, promotes insulin secretion, suppresses glucagon secretion; targets the vagus nerve, hindbrain, and hypothalamus	4^(ref. 127)^ (63 RCTs)
Insulin	Pancreatic β cells	Targets the hypothalamus	4 (4 systematic reviews^127, 128, 129, 130^) (136 RCTs)
Leptin	White adipose tissue, stomach (secreted in proportion to adipose volume and total fat mass)	Signals the brainstem and arcuate nucleus of the hypothalamus when fat stores are low	2^(ref. 86)^ (68 RCTs)
Neuromedin B (NMB)	Gastric myenteric neurons	Contributes to meal termination	0^(ref. 131)^
Neurotensin	Gastrointestinal enteroendocrine cells	Decrease in food intake is acute only	1^(ref. 132)^
Obestatin	Stomach, intestine	Decreases food intake, slows gastric emptying	2^(ref. 74)^ (2 RCTs)
Oxyntomodulin (OXM)	Intestinal L cells (co-secreted with GLP-1, PYY)	Delays gastric emptying; targets the hypothalamus	2^(ref. 133)^ (3 RCTs)
Oxytocin	Hypothalamus	Projections to caudal brainstem; inhibits gastric emptying	2^(ref. 134)^ (1 RCT)
Pancreatic polypeptide (PP)	Pancreatic F cells	Delays gastric emptying; targets the vagus nerve and hindbrain	2^(ref. 42)^ (8 RCTs)
Peptide tyrosine-tyrosine (PYY)	Intestinal L cells (co-secreted with GLP-1, OXM)	Delays gastric emptying; targets the vagus nerve and hypothalamus	2^(ref. 135)^ (50 RCTs)
Serotonin	Midbrain, hindbrain	Targets hypothalamus	4^(ref. 136)^ (21 RCTs)
Vasoactive intestinal polypeptide (VIP)	Intestine, pancreas	Targets hypothalamus	1^(ref. 137)^
			
*Orexigenic factors*			
Agouti-related peptide (AgRP)	Hypothalamus	MC3/4 receptor antagonist	2^(ref. 138)^ (2 RCTs)
Endocannabinoids	Hypothalamus	Cannabinoid receptors widely distributed throughout the CNS	2^(ref. 139)^ (2 RCTs)
γ-aminobutyric acid (GABA)	Ubiquitous in CNS	GABA/AgRP co-expressing neurons targeting the parabrachial nucleus of the hypothalamus	0^(ref. 140)^
Galanin	Widely distributed in CNS, PNS and gut	Central stimulation of food intake mediated by galanin receptor-1; modulates hippocampal circuits	2^(ref. 141)^ (1 RCT)
Ghrelin	Gastric antrum and fundus	Increases gastric emptying, decreases insulin secretion; targets the vagus nerve, hindbrain and hypothalamus	2^(ref. 142)^ (114 RCTs)
Glutamate	Ubiquitous in CNS	Stimulates AgRP/NPY-expressing neurons in the hypothalamus	1^(ref. 143)^
Melanin-concentrating hormone (MCH)	Hypothalamus	MCH neurons have widespread projections throughout the brain	1^(ref. 144)^
Motilin	Small intestine	Increases gastric motility, targets vagus nerve	1^(ref. 145)^
Neuropeptide W (NPW)	Hypothalamus, stomach	Orexigenic: activation of melanin-concentrating hormone- and orexin-containing neurons	0^(ref. 146)^
Neuropeptide Y (NPY)	Hypothalamus	Stimulates Y1 and Y5 receptors; modulates hypothalamic circuits	2^(ref. 141)^ (5 RCTs)
Orexin (hypocretin)	Central: Hypothalamus Periphery: enteric plexus, mucous and musculature in the gut	Stimulates gastric emptying	1^(ref. 147)^
Visfatin	Visceral adipose tissue	Potentially modulates hypothalamic circuits	1^(ref. 148)^

Abbreviations: CNS, central nervous system; MC, melanocortin; PNS, peripheral nervous system; RCT, randomized controlled trial.

0=animal studies only; 1=observational clinical studies; 2=randomized controlled trials; 3=systematic reviews of observational studies; 4=systematic reviews of randomized controlled trials (with or without observational studies as well).
