# Supplementary material for: Altered gut and adipose tissue hormones in overweight and obese individuals: cause or consequence?
Source: Int J Obes (Lond). 2015 Dec 1;40(4):622–32. doi: 10.1038/ijo.2015.220 (PMC4827002; doi:10.1038/ijo.2015.220)
Supplement: Supplementary Appendix 1 [file ijo2015220x1.pdf]

Altered Gut and Adipose Tissue Hormones in Overweight and Obese Individuals: Cause or Consequence?

Authors: Michael E.J. Lean, MA, MB, BChir, MD (Cantab), FRCP (Edin), FRCPS (Glas) *; Dalia Malkova, PhD

Supplementary Information: Appendix 1

Summary

This supplementary information to the manuscript entitled 'Altered Gut and Adipose Tissue Hormones in Overweight and Obese Individuals: Cause or Consequence?' describes the effect of gastric bypass surgery on intestinal satiety hormones.

Effect of Gastric Bypass Surgery on Intestinal Satiety Hormones

Gastric bypass surgery results in significant and well- (but seldom completely-) sustained weight loss in severely obese individuals.[1] It has been postulated that hormonal regulation of appetite may be altered following surgery, particularly with bypass procedures, and that this alteration may assist post-surgical weight control.[2-4]

One of the most popular procedures at present is Roux-en-Y gastric bypass (RYGB) surgery, a complex intervention that reduces the size of the stomach and circumvents the duodenum, to deliver ingesta directly to the small intestine. There, its rapid delivery has various adverse effects, such as rapid glucose and amino acid absorption, which may generate hyperinsulinism and subsequent unpleasant or even dangerous hypoglycaemia, known as 'dumping syndrome'. Complicating matters, there are also profound osmotic effects with consequent sympathetic nervous system and catecholamine stimulation.[5] It has been argued that excessively rapid delivery of ingesta to the jejunum may promote increased postprandial GLP-1 secretion, thereby contributing to increased satiety signalling and decreased food

intake.[4, 6, 7] However, the evidence for this is weak. In weight-reduced patients (mean BMI, 35.6 kg/m^2) a mean of 43 months after RYGB surgery, greater postprandial concentrations of GLP-1 and PYY were observed 30 minutes after a standard meal compared with severely obese control subjects (mean BMI, 44.3 kg/m^2).[8] This study did not explicitly state whether or not subjects were weight-stable when gut hormones were measured, and there were no observations on subjects with non-surgical weight loss of the same degree. Any effects from the physical consequences of surgery, with altered food presentation to gut mucosa, cannot be differentiated from the metabolic consequences of marked negative energy balance. The relationship between subjective appetite ratings and gut hormones was examined in 12 obese patients before and 1 year after RYGB surgery by Bryant et al.[9] At follow-up, concentrations of postprandial GLP-1 were greater, while self-reported hunger and the desire to eat decreased and fullness increased. Fasting GLP-1 levels positively correlated with fullness and negatively correlated with the desire to eat. This proposed mechanism therefore seems plausible; however, estimation of appetite and satiety is imprecise, and it is not possible to be sure that it has any quantitative impact beyond those of severe energy restriction and weight loss in this complicated human model.

The time course of changes in satiety hormones was examined in 12 severely obese subjects (mean baseline BMI, 45.3 kg/m^2) who consumed a 300-kcal liquid test meal pre-operatively and at 3 days, 2 months, and 1 year after RYGB surgery.[10] Postprandial GLP-1 concentrations (**Figure 1**) and the 3-hour AUC ($AUC_{0\text{-}180\ min}$) progressively increased over the 1-year period. Plasma levels of ghrelin decreased on Day 3, but returned to pre-operative baseline levels thereafter. No immediate changes in leptin concentrations occurred at Day 3, but levels were significantly lower after 2 months and these levels were sustained at 1 year. Visual analogue scale ratings of fullness significantly increased and hunger ratings significantly decreased as

early as postoperative Day 3 and were sustained at 1 year.[10] Data from another study demonstrated that increased postprandial GLP-1 and PYY responses to a test meal corresponded with sustained increases in subjective satiety ratings compared with pre-operative baseline responses in patients' status 1 to 2 years after RYGB surgery.[11] Again, the lack of a non-surgical weight loss group makes interpretation of these findings difficult.

Figure 1. Plasma levels of glucagon-like peptide-1 (GLP-1) after a test meal consumed prior to and 3 days, 2 months, and 1 year after Roux-en-Y gastric bypass surgery in 12 obese patients.[10] Reproduced with permission.

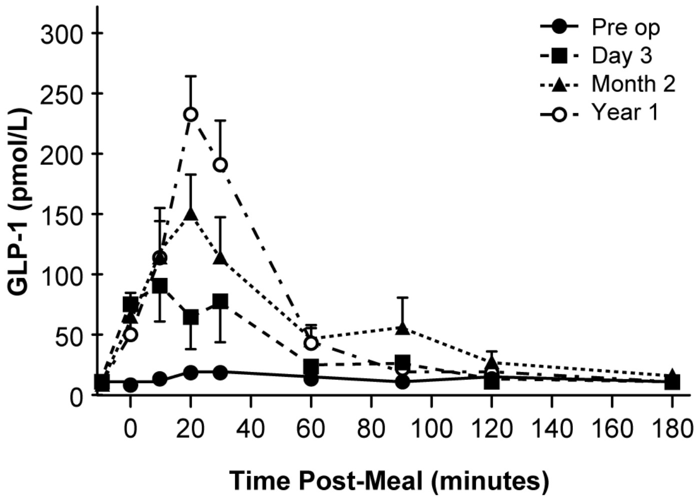

To elucidate mechanisms underlying altered hormonal signalling, Dirksen and colleagues attempted to establish whether alterations in food transit through the gastrointestinal tract modulated gut hormone release after RYGB surgery.[12] Exaggerated postprandial secretion of GLP-1 and PYY during multiple meal tests was reported in obese patients who had undergone RYGB surgery at least 1 year prior to the study compared with lean control subjects. Gastric pouch emptying was rapid and transit through the small intestine was slower in patients after RYGB surgery compared with control subjects. Faster emptying of the gastric pouch in patients with RYGB surgery appeared to be associated with increased gut hormone

concentrations. The relationship between gut hormone changes and weight-loss response was further assessed in obese patients who had undergone RYGB surgery at least 1 year earlier.[13] GLP-1 release and ghrelin suppression were significantly enhanced in patients who experienced >60% loss of excess BMI compared with those who had <50% loss. The gut hormone profile in patients with a greater weight loss response could suggest that alterations in gut hormones may promote weight loss, or maintenance of weight loss, after RYGB surgery. However, reverse causality remains possible: greater weight losses may have had greater effects on gut hormones. Similarly, 6 years after patients underwent RYGB surgery or laparoscopic adjustable gastric banding (LAGB) surgery, patients in the RYGB group maintained greater loss of weight and fat mass, as well as enhanced postprandial responses of GLP-1 and PYY, compared with patients in the LABG group.[14]

An alternative explanation might be that patients with greater weight losses engaged in post-operative weight-control behaviours that had secondary effects which elevated gut satiety hormones. For instance, in a randomized controlled trial of patients who had undergone RYGB surgery 6 months previously, a comprehensive diet and exercise intervention resulted in significantly greater levels of physical activity accompanied by greater weight loss 1 year after RYGB surgery compared with patients who received only brief guidelines for healthy eating and physical activity.[15] This finding is in line with the evidence presented in the above text discussing changes of gut hormones with exercise.

Another study demonstrated enhanced secretion of GLP-1 and PYY after RYGB surgery; brain magnetic resonance imaging (MRI) suggested that the hedonic activation of brain reward systems was decreased in patients who underwent RYGB surgery compared with those who had undergone LAGB surgery.[16] This suggests that greater weight loss after RYGB

surgery may be mediated in part by anorexigenic peptides regulating brain reward centres; however, the data were complicated by more frequent dumping syndrome and nausea in patients who underwent RYGB surgery. Interpretation of such studies may also be complicated by selection criteria, and different peri- and post-operative courses.

Many factors therefore contribute to weight loss and gut hormone profiles after bariatric surgery; thus, causal relationships remain unclear. Overall, the results of most of the studies outlined above are consistent in supporting a plausible mechanism such that the relationship between GLP-1, PYY, ghrelin and appetite may contribute to a reduced tendency to overeat after RYGB surgery. However, many other factors may mediate this apparent relationship, including hyperinsulinism, dumping syndrome, subclinical osmotic effects and elevated bile acid concentrations. Appetite hormones are probably not the dominant factor for improved glucose tolerance after gastric surgery, and indeed the role of appetite hormones is put in doubt by an important study of 140 patients by Schauer et al, who compared Roux-en-Y with sleeve gastrectomy.[17] They reported almost equal reductions in glycated haemoglobin, with almost equal, substantial, weight losses. There was no added incretin effect from the Roux-en-Y operation (**Figure 2**). The influence of other appetite-regulating hormones on sustained weight loss following gastric bypass surgery requires further study.

Figure 2. Change in glucose control and BMI after sleeve gastrectomy compared with Roux-en-Y bypass.[17] Reproduced with permission.

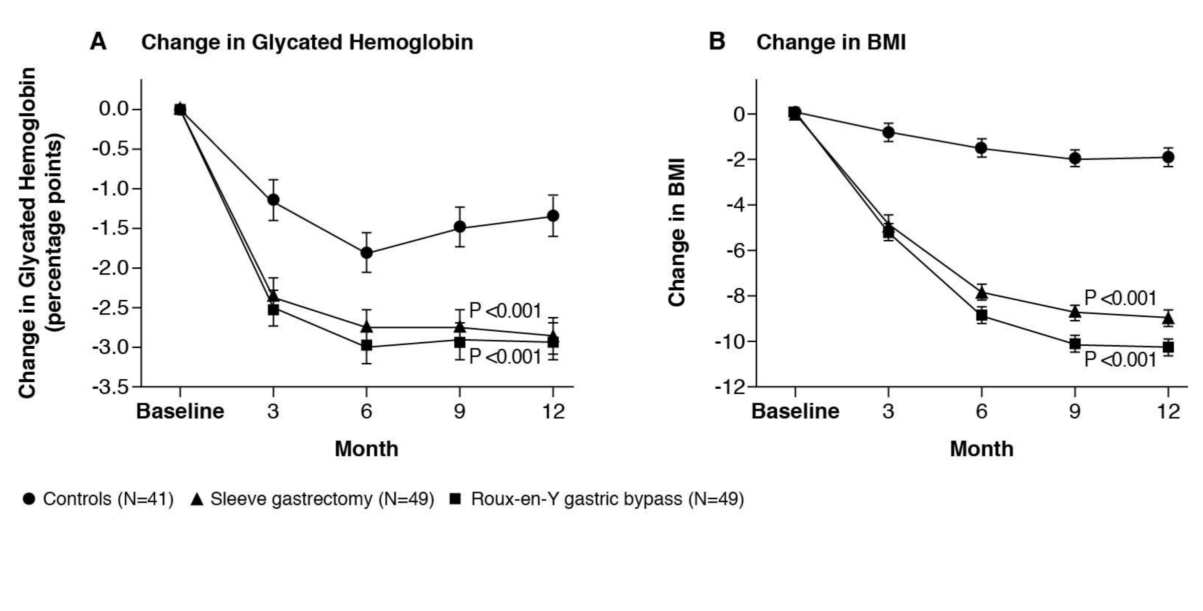

References

1. Sjostrom L. Review of the key results from the Swedish Obese Subjects (SOS) trial - a prospective controlled intervention study of bariatric surgery. *J Intern Med* 2013; **273**: 219-234.
2. Cummings DE, Weigle DS, Frayo RS, Breen PA, Ma MK, Dellinger EP, *et al.* Plasma ghrelin levels after diet-induced weight loss or gastric bypass surgery. *N Engl J Med* 2002; **346**: 1623-1630.
3. Jayasena CN, Bloom SR. Role of gut hormones in obesity. *Endocrinol Metab Clin North Am* 2008; **37**: 769-787, xi.
4. Ochner CN, Gibson C, Carnell S, Dambkowski C, Geliebter A. The neurohormonal regulation of energy intake in relation to bariatric surgery for obesity. *Physiol Behav* 2010; **100**: 549-559.
5. Ponsky TA, Brody F, Pucci E. Alterations in gastrointestinal physiology after Roux-en-Y gastric bypass. *J Am Coll Surg* 2005; **201**: 125-131.
6. Kashyap SR, Daud S, Kelly KR, Gastaldelli A, Win H, Brethauer S, *et al.* Acute effects of gastric bypass versus gastric restrictive surgery on beta-cell function and insulinotropic hormones in severely obese patients with type 2 diabetes. *Int J Obes* 2010; **34**: 462-471.
7. Korner J, Inabnet W, Febres G, Conwell IM, McMahon DJ, Salas R, *et al.* Prospective study of gut hormone and metabolic changes after adjustable gastric banding and Roux-en-Y gastric bypass. *Int J Obes* 2009; **33**: 786-795.
8. Holdstock C, Zethelius B, Sundbom M, Karlsson FA, Eden Engstrom B. Postprandial changes in gut regulatory peptides in gastric bypass patients. *Int J Obes* 2008; **32**: 1640-1646.
9. Bryant EJ, King NA, Falken Y, Hellstrom PM, Holst JJ, Blundell JE, *et al.* Relationships among tonic and episodic aspects of motivation to eat, gut peptides, and weight before and after bariatric surgery. *Surg Obes Relat Dis* 2013; **9**: 802-808.
10. Falken Y, Hellstrom PM, Holst JJ, Naslund E. Changes in glucose homeostasis after Roux-en-Y gastric bypass surgery for obesity at day three, two months, and one year after surgery: role of gut peptides. *J Clin Endocrinol Metab* 2011; **96**: 2227-2235.
11. Pournaras DJ, Osborne A, Hawkins SC, Mahon D, Ghatei MA, Bloom SR, *et al.* The gut hormone response following Roux-en-Y gastric bypass: cross-sectional and prospective study. *Obes Surg* 2010; **20**: 56-60.
12. Dirksen C, Damgaard M, Bojsen-Moller KN, Jorgensen NB, Kielgast U, Jacobsen SH, *et al.* Fast pouch emptying, delayed small intestinal transit, and exaggerated gut hormone responses after Roux-en-Y gastric bypass. *Neurogastroenterol Motil* 2013; **25**: 346-351+e255.
13. Dirksen C, Jorgensen NB, Bojsen-Moller KN, Kielgast U, Jacobsen SH, Clausen TR, *et al.* Gut hormones, early dumping and resting energy expenditure in patients with good and poor weight loss response after Roux-en-Y gastric bypass. *Int J Obes (Lond)* 2013; **37**: 1452-1459.
14. Werling M, Fandriks L, Bjorklund P, Maleckas A, Brandberg J, Lonroth H, *et al.* Long-term results of a randomized clinical trial comparing Roux-en-Y gastric bypass with vertical banded gastroplasty. *Br J Surg* 2013; **100**: 222-230.
15. Nijamkin MP, Campa A, Sosa J, Baum M, Himburg S, Johnson P. Comprehensive nutrition and lifestyle education improves weight loss and physical activity in Hispanic Americans following gastric bypass surgery: a randomized controlled trial. *J Acad Nutr Diet* 2012; **112**: 382-390.
16. Scholtz S, Miras AD, Chhina N, Prechtl CG, Sleeth ML, Daud NM, *et al.* Obese patients after gastric bypass surgery have lower brain-hedonic responses to food than after gastric banding. *Gut* 2014; **63**: 891-902.
17. Schauer PR, Kashyap SR, Wolski K, Brethauer SA, Kirwan JP, Pothier CE, *et al.* Bariatric surgery versus intensive medical therapy in obese patients with diabetes. *N Engl J Med* 2012; **366**: 1567-1576.
