# Supplementary material for: Altered gut and adipose tissue hormones in overweight and obese individuals: cause or consequence?
Source: Int J Obes (Lond). 2015 Dec 1;40(4):622–32. doi: 10.1038/ijo.2015.220 (PMC4827002; doi:10.1038/ijo.2015.220)
Supplement: Supplementary Appendix 2 [file ijo2015220x2.pdf]

Altered Gut and Adipose Tissue Hormones in Overweight and Obese Individuals: Cause or Consequence?

Authors: Michael E.J. Lean, MA, MB, BChir, MD (Cantab), FRCP (Edin), FRCPS (Glas) *; Dalia Malkova, PhD

Supplementary Information: Appendix 2

Summary

This supplementary information to the manuscript entitled 'Altered Gut and Adipose Tissue Hormones in Overweight and Obese Individuals: Cause or Consequence?' describes brain activity in relation to obesity and satiety hormones.

Obesity, Satiety Hormones, and Brain Activity

Neuroimaging studies illustrate neuroanatomical and functional correlates of satiety and eating behaviours in obesity.[1] Using functional MRI (fMRI), Matsuda and colleagues demonstrated the hypothalamic responses to a single oral glucose load in obese and lean individuals following a 12-hour fast. Compared with lean subjects, the hypothalamic regions in obese persons showed blunted and delayed inhibitory responses to glucose challenge. Fasting glucose and insulin concentrations correlated positively with delays in hypothalamic responses.[2]

DelParigi and co-workers used positron emission tomography (PET) imaging to measure the effect of the sensory experience of food on brain activation during extreme hunger. Following a 36-hour fast, obese and lean subjects consumed a test meal (i.e., to simulate tasting, not eating) and then underwent PET scanning. Obese subjects had a significantly greater response in sensory areas of the brain (i.e., middle-dorsal insular cortex) than lean subjects. Moreover,

responses in obese subjects correlated with the degree of obesity and blood glucose levels.[3] Other imaging studies using PET scans and fMRI showed that obesity was associated with satiation-mediated changes in activity in regions of the prefrontal cortex.[4, 5]

Studies showing the effects of a test meal on correlations between postprandial gut hormone levels and patterns of brain activation are limited. One study measured postprandial levels of GLP-1 and brain activation using PET scans and fMRI in healthy volunteers (mean BMI, 31 kg/m^2; range, 18.5–50 kg/m^2) who underwent a 36-hour fast followed by consumption of a liquid meal, which provided 50% of the subject’s resting energy expenditure and resulted in satiation (hunger and fullness were measured using 100-mm visual analogue scales).[6] Changes in GLP-1 levels following the test meal correlated significantly with activation in the left dorsolateral prefrontal cortex and the hypothalamus; these changes were independent of age, sex, adiposity, and meal-related changes in insulin, glucose, and free fatty acid levels. In another study, activity in several reward regions of the brain in response to food images was measured using blood oxygen level–dependent fMRI.[7] In healthy, normal-weight participants, infusion of PYY and GLP-1 in the fasted state decreased activity in brain reward regions similarly to the response observed with feeding.

Differences between obese and lean subjects in the neuronal response to food cues have been observed in neuroimaging studies.[8, 9] In one study, activity in mesolimbic reward regions in response to hedonic visual food cues in obese and non-obese control subjects was assessed using fMRI.[8] Food cue–induced activity in the ventral striatum was significantly associated

with BMI and with leptin concentration in plasma, suggesting that leptin plays a role in modulating activity in mesolimbic circuits in response to food cues.

Neuronal activity in subjects who identified themselves as obese-prone or obese-resistant in response to food cues was assessed by fMRI in another study.[9] Obese-prone subjects indicated that they struggled with weight management, had a history of weight fluctuations but were currently weight stable, had a BMI between 20 kg/m^2 and 30 kg/m^2, and had at least one obese first-degree relative. Obese-resistant subjects defined themselves as naturally thin, with a BMI of 17 to 25 kg/m^2, expending little energy to remain thin, and reported no obese first-degree relatives. Comparative fMRI imaging was performed while participants viewed food or non-food images during fasted and fed states. Food cues elicited responses in the insula, parietal cortex, somatosensory cortex, and visual cortex in both subject groups in the fasted state; feeding significantly attenuated activity in these regions in obese-resistant but not obese-prone subjects. The test meal also resulted in greater activity in the medial and anterior prefrontal cortex of obese-prone subjects compared with obese-resistant subjects. These results suggest that food cue–induced activity in brain regions involved in energy intake is altered in individuals prone to obesity, compared with individuals who have always been lean.

Recent evidence suggests that brain responses to food stimuli, particularly within reward processing and appetite-related regions, are favourably modified by participation in exercise programmes.[10, 11] Cornier reported that exercise training for 6 months reduced responses to visual stimuli of food measured in the fasted state and that reduction in the neuronal response to visual food cues food was positively correlated with change in fat/body mass.[10] Another

study reported an association between the total number of minutes of self-reported weekly physical exercise and reduced activation in food-responsive reward regions, which in turn was associated with reduced preference ratings specific for high-calorie foods particularly those with a savoury flavour.[11] It remains unclear whether changes in the neural response induced by exercise training have a direct impact on eating behaviours and appetite.[10]

Studies reporting the investigation of mechanisms by which exercise modifies neural responses to images of food stimuli during exercise interventions are scarce. A study in normal weight men found that exercise session-induced changes in neural responses in reward related regions of the brain in response to food have been associated with significant increases in PYY concentrations and reductions in total and acylated ghrelin concentrations.[12] Further support for a role of ghrelin in modulation of neurological responses to food is available from a recent study by Schellekens.[13] Data obtained by Cornier et al.[10] suggest that the modification of response to visual food stimuli with chronic exercise may also be related to potential improvement in sensitivity to leptin, a hormone known to impact higher brain responses.[14] Consistent with other reports on the reduction in plasma leptin levels following 3–4 months of aerobic exercise,[15, 16] the neuroimaging study of Cornier et al.[10] found that 6 months' participation in physical activity induced significant reduction in plasma leptin concentrations. Despite this, activity of brain regions important in motivation to eat was reduced, and no increase in hunger or drive to eat was found,[10] indicating increased sensitivity to leptin.

References

1. Gibson CD, Carnell S, Ochner CN, Geliebter A. Neuroimaging, gut peptides and obesity: novel studies of the neurobiology of appetite. *J Neuroendocrinol* 2010; **22**: 833-845.
2. Matsuda M, Liu Y, Mahankali S, Pu Y, Mahankali A, Wang J*, et al.* Altered hypothalamic function in response to glucose ingestion in obese humans. *Diabetes* 1999; **48**: 1801-1806.
3. DelParigi A, Chen K, Salbe AD, Reiman EM, Tataranni PA. Sensory experience of food and obesity: a positron emission tomography study of the brain regions affected by tasting a liquid meal after a prolonged fast. *Neuroimage* 2005; **24**: 436-443.
4. Gautier JF, Del Parigi A, Chen K, Salbe AD, Bandy D, Pratley RE*, et al.* Effect of satiation on brain activity in obese and lean women. *Obes Res* 2001; **9**: 676-684.
5. Le DS, Chen K, Pannacciulli N, Gluck M, Reiman EM, Krakoff J. Reanalysis of the obesity-related attenuation in the left dorsolateral prefrontal cortex response to a satiating meal using gyral regions-of-interest. *J Am Coll Nutr* 2009; **28**: 667-673.
6. Pannacciulli N, Le DS, Salbe AD, Chen K, Reiman EM, Tataranni PA*, et al.* Postprandial glucagon-like peptide-1 (GLP-1) response is positively associated with changes in neuronal activity of brain areas implicated in satiety and food intake regulation in humans. *Neuroimage* 2007; **35**: 511-517.
7. De Silva A, Salem V, Long CJ, Makwana A, Newbould RD, Rabiner EA*, et al.* The gut hormones PYY 3-36 and GLP-1 7-36 amide reduce food intake and modulate brain activity in appetite centers in humans. *Cell Metab* 2011; **14**: 700-706.
8. Grosshans M, Vollmert C, Vollstadt-Klein S, Tost H, Leber S, Bach P*, et al.* Association of leptin with food cue-induced activation in human reward pathways. *Arch Gen Psychiatry* 2012; **69**: 529-537.
9. Cornier MA, McFadden KL, Thomas EA, Bechtell JL, Eichman LS, Bessesen DH*, et al.* Differences in the neuronal response to food in obesity-resistant as compared to obesity-prone individuals. *Physiology and Behavior* 2013; **110-111**: 122-128.
10. Cornier MA, Melanson EL, Salzberg AK, Bechtell JL, Tregellas JR. The effects of exercise on the neuronal response to food cues. *Physiol Behav* 2012; **105**: 1028-1034.
11. Killgore WD, Kipman M, Schwab ZJ, Tkachenko O, Preer L, Gogel H*, et al.* Physical exercise and brain responses to images of high-calorie food. *Neuroreport* 2013; **24**: 962-967.
12. Crabtree DR, Chambers ES, Hardwick RM, Blannin AK. The effects of high-intensity exercise on neural responses to images of food. *Am J Clin Nutr* 2014; **99**: 258-267.
13. Schellekens H, Dinan TG, Cryan JF. Ghrelin at the interface of obesity and reward. *Vitam Horm* 2013; **91**: 285-323.
14. Baicy K, London ED, Monterosso J, Wong ML, Delibasi T, Sharma A*, et al.* Leptin replacement alters brain response to food cues in genetically leptin-deficient adults. *Proc Natl Acad Sci U S A* 2007; **104**: 18276-18279.
15. Polak J, Klimcakova E, Moro C, Viguerie N, Berlan M, Hejnova J*, et al.* Effect of aerobic training on plasma levels and subcutaneous abdominal adipose tissue gene expression of adiponectin, leptin, interleukin 6, and tumor necrosis factor alpha in obese women. *Metabolism* 2006; **55**: 1375-1381.
16. Pasman WJ, Westerterp-Plantenga MS, Saris WH. The effect of exercise training on leptin levels in obese males. *Am J Physiol* 1998; **274**: E280-286.
